# Intervention Costs From Communities Putting Prevention to Work

**DOI:** 10.5888/pcd13.150368

**Published:** 2016-07-28

**Authors:** Amanda A. Honeycutt, Olga A. Khavjou, Christina Bradley, Simon Neuwahl, Thomas J. Hoerger, David Bellard, Amanda J. Cash

**Affiliations:** Author Affiliations: Amanda A. Honeycutt, Olga A. Khavjou, Christina Bradley, Simon Neuwahl, Thomas J. Hoerger, David Bellard, RTI International, Research Triangle Park, North Carolina; Amanda J. Cash, US Department of Health and Human Services, Office of the Assistant Secretary for Planning and Evaluation, Washington, DC.

## Abstract

**Introduction:**

In 2010, the Centers for Disease Control and Prevention funded 50 communities to participate in the Communities Putting Prevention to Work (CPPW) program. CPPW supported community-based approaches to prevent or delay chronic disease and promote wellness by reducing tobacco use and obesity. We collected the direct costs of CPPW for the 44 communities funded through the American Recovery and Reinvestment Act (ARRA) and analyzed costs per person reached for all CPPW interventions and by intervention category.

**Methods:**

From 2011 through 2013, we collected quarterly data on costs from the 44 CPPW ARRA-funded communities. We estimated CPPW program costs as spending on labor; consultants; materials, travel, and services; overhead activities; and partners plus the value of in-kind donations. We estimated communities’ costs per person reached for each intervention implemented and compared cost allocations across communities that focused on reducing tobacco use, or obesity, or both. Analyses were conducted in 2014; costs are reported in 2012 dollars.

**Results:**

The largest share of CPPW total costs of $363 million supported interventions in communities that focused on obesity ($228 million). Average costs per person reached were less than $5 for 84% of tobacco-related interventions, 88% of nutrition interventions, and 89% of physical activity interventions. Costs per person reached were highest for social support and services interventions, almost $3 for tobacco‑use interventions and $1 for obesity prevention interventions.

**Conclusions:**

CPPW cost estimates are useful for comparing intervention cost per person reached with health outcomes and for addressing how community health intervention costs vary by type of intervention and by community size.

## Introduction

Preventing chronic disease and its associated illness, death, and cost requires a comprehensive approach that includes population-wide approaches to make healthful living easier (1). In 2010, the US Department of Health and Human Services launched the 2-year Communities Putting Prevention to Work (CPPW) initiative to support policies, systems, and environmental changes to prevent obesity, reduce tobacco use or exposure to secondhand smoke, or reduce both obesity and tobacco use (2,3). The 2009 American Recovery and Reinvestment Act (ARRA) funded 44 of the 50 CPPW communities. The CPPW cost evaluation, which was also funded under ARRA, covered only the 44 ARRA-funded communities; the 6 communities that were later funded under the Affordable Care Act were not covered in the cost evaluation.

The ARRA-funded CPPW awards were intended to reduce tobacco use and exposure to secondhand smoke in 14 communities, prevent obesity in 23 communities, and prevent both tobacco use and obesity in 7 communities. These awardees consisted of 14 large cities, 11 urban areas, 16 small cities or rural counties, and 3 tribal nations.

Our study’s purpose was to analyze CPPW program costs as part of a multicomponent evaluation (3–5). We used a prospective cost data collection approach, systematically applied across the 44 communities, and an instrument developed specifically for CPPW (6). Our prospective data collection approach was novel and intended to limit recall bias; previous analyses collected and analyzed community-based program costs retrospectively by interviewing program administrators after the programs had ended (7,8). CPPW cost estimates will be useful to communities that plan to implement similar interventions and for evaluations that compare outcomes with costs.

## Methods

### Cost data collection

We collected CPPW cost data quarterly from the 44 ARRA-funded communities via a web-based Cost Study Instrument (CSI) (6). We provided each community with access to the CSI to report quarterly costs for the program award period (2010 through 2013); the first reporting period covered the first year of funding. We obtained cost data for 1) labor, 2) consultants, 3) materials, travel, and services, 4) overhead activities (ie, indirect expenditures), and 5) partner organization work (ie, contracted services). In addition to actual expenditures, we collected information on voluntary or in-kind contributions from communities and their partners.

Each community developed a CPPW community action plan that included multiple community-defined objectives to improve population health. We asked communities to report quarterly costs for each resource category (eg, labor) and enter the percentage of costs allocated to each CPPW objective. Because objectives were unique to each community, we also asked communities to indicate which of a common set of CPPW strategies supported each objective. CPPW strategies were collectively known as media, access, point of promotion/decision, price, and social support and services (MAPPS). Each MAPPS strategy could have supported multiple community objectives, and each objective had one or more MAPPS strategies linked to it. A media strategy was hard-hitting counteradvertising for tobacco; an access strategy was limiting availability of unhealthy food and drink.

### Cost data aggregation

We estimated community costs at the most disaggregated level by generating objective- and MAPPS strategy-level costs. For example, if a community had 10 objectives, and each objective had 2 MAPPS strategies linked to it, we estimated costs for 20 objective and strategy combinations. Communities also allocated costs to administration or evaluation. We assigned administrative costs to program cost estimates in proportion to spending on each objective or strategy. We also estimated evaluation costs but excluded them from program cost estimates.

CPPW interventions were defined after the programs were under way and were usually more specific than MAPPS strategies. For example, “healthy vending” was an intervention linked to the broader MAPPS strategy “healthy food/drink availability.” We estimated CPPW intervention costs by assigning objective/strategy costs to each intervention that was linked to a community objective. 

### Cost analyses

First, we calculated each awardee’s total CPPW spending and compared it with the total value of its CPPW award to validate awardees’ reported spending. “Spending” is the actual dollar outlays for CPPW, which included evaluation expenditures. We next estimated total CPPW program costs as the sum of spending on labor; materials, travel, and supplies; overhead; and partners, but excluding evaluation costs because such costs are for research and do not reflect costs to deliver CPPW interventions. Grantees were required to evaluate their programs, but without specific guidance on how much to spend. We then added the value of in-kind donations to ensure that our costs reflect the full value of resources used for CPPW. We used CPPW program cost estimates (excluding evaluation costs, but including in-kind contributions) in all cost analyses.

We analyzed costs from the provider’s perspective, excluding costs incurred by program participants. Analyses were conducted in 2014 using costs collected from 2011 to 2013, which covered the full period of CPPW (2010–2013). We adjusted costs to 2012 dollars using gross domestic product price indices (9). Any costs incurred in 2013 were for a limited number of communities in the first quarter and were treated as 2012 costs. We estimated costs at multiple levels (total, community, and intervention) and for resource and MAPPS categories.

Each intervention could have supported multiple objectives. We estimated community intervention costs as the sum of all objective costs assigned to an intervention. For example, if the intervention “Create safe places for physical activity” was used to support 3 community objectives and no other interventions supported those objectives, then we summed the costs for those 3 objectives to estimate the intervention cost. If multiple interventions supported an objective, we calculated intervention costs by weighting the relevant objective costs (eg, including one-half of costs for objectives supported by 2 interventions).

We summed community intervention costs across all communities to estimate aggregate costs for each CPPW intervention. To estimate the cost per person reached, we needed estimates of the number of people reached by each intervention. Because data on reach was not collected at the intervention level, we estimated intervention reach using objective reach data that communities submitted to the Centers for Disease Control and Prevention (CDC). These data were supplemented with narratives on program accomplishments written by CDC program staff, then reviewed and validated by CPPW program officers, subject matter experts, and evaluation contractors. Reviewers used US Census Bureau, school, and other local data sources to validate reach estimates. If an intervention was linked to only one objective, we assumed intervention reach was equal to objective reach. If an intervention was linked to multiple objectives, we used the maximum objective reach to approximate intervention reach. Objectives were marked as complete or incomplete by the end of the grant period. We used an estimate of “interim reach” for incomplete objectives, but we dropped interventions for which data on interim reach were not available. Of the 768 community interventions for which we collected cost data, 120 were eliminated because of missing reach data; costs assigned to the dropped interventions totaled $33 million.

We calculated intervention costs per person reached as aggregate intervention costs across implementing communities divided by aggregate intervention reach. We also averaged community intervention costs per person reached, thus giving equal weight to costs regardless of community size. In all analyses, a community was considered to have worked on an intervention only if it had non-zero costs and if intervention reach could be estimated from objective reach data. We also averaged intervention costs by MAPPS category.

We plotted CPPW intervention costs per person reached against intervention reach to examine whether per person costs declined as the number of people reached increased and to explore differences by community type. Community types were tribal, state-coordinated, urban, or large city, where state-coordinated communities supported work in 2 separate cities or rural areas of up to 500,000 people, urban areas had populations of 500,000 to 1 million, and large cities had populations of more than 1 million. 

## Results

The award amount for the 44 CPPW ARRA communities was $379 million. Total spending for these communities was $376 million; of that, $33 million was for evaluation. Total CPPW program costs, which are equal to total payments minus the amount for evaluation ($33 million) plus the value of in-kind costs ($21 million), were $363 million — $136 million for tobacco and $228 million for obesity prevention. CPPW communities spent an average of 9% of their total awards (ranging from 3% to 18%) on evaluations.

CPPW partner costs were the largest share of total costs (52%) (data not shown). The next largest share was for labor (20%), followed by materials, travel, and services (12%); administration (7%); and consultant expenditures (3%). Communities that focused on obesity had a higher percentage of in-kind contributions (7%) than communities that focused on tobacco (4%). Communities that focused on tobacco had a higher share of costs for materials, travel, and services (15%) than communities that focused on obesity (9%), probably reflecting purchase of tobacco use cessation services and nicotine replacement therapy.

By analyzing tobacco intervention costs per person reached, we estimated costs of $1.02 per person reached for “usage bans,” but costs varied from $0.16 to $115.37 across the 20 communities that implemented this intervention (Table 1). The tobacco intervention “Hard-hitting counteradvertising” had average costs of $1.57 per person reached; costs were similar across the 14 communities with reach data. The highest cost tobacco intervention was “Cessation services, other,” with a mean cost per person reached of $5.14. Costs for this intervention varied across the 10 communities with reach data, ranging from $0.33 to $7,757.20 per person reached; the smallest communities had the highest costs. We report estimates of total costs and costs per person reached for the most widely used tobacco, nutrition, and physical activity interventions (Table 1). Appendices A and B provide cost estimates for all tobacco interventions.

**Table 1:** Summary of Total Costs, Reach, and Costs per Person Reached for Key CPPW Interventions, 2012 US Dollars, 44 US Communities, 2010–2013

Intervention Description	N	Total Costs			Costs Per Person Reached		
		Aggregate Cost, $	Mean Community Cost, $	Total No. of People Reached	Weighted Mean^a^ (SD), $	Minimum, $	Maximum, $
**Tobacco**							
Usage bans	20	26,566,358	1,328,318	25,976,876	1.02 (26.25	0.16	115.37
Hard-hitting counteradvertising	14	35,433,968	2,530,998	22,550,786	1.57 (3.78)	0.16	14.11
Cessation services — other	10	11,190,978	1,119,098	2,175,929	5.14 (2,448.29)	0.33	7,757.20
**Nutrition**							
Media to support improved nutrition to prevent obesity	27	29,132,936	1,078,998	23,033,492	1.26 (2.77)	0.33	10.14
Restrict availability of less healthy foods and beverages	22	11,594,192	527,009	20,988,404	0.55 (14.67)	0.10	64.46
Enhance access to healthy food retailer or healthier retail food, not transportation	22	8,890,749	404,125	15,311,544	0.58 (2.42)	0.03	8.24
**Physical activity**							
Media to support improved physical activity to prevent obesity	28	24,482,982	874,392	32,417,636	0.76 (525.08)	0.17	2,783.74
Environmental supports to promote walking and cycling and other physical activity	20	13,025,401	651,270	23,178,552	0.56 (124.87)	0.02	461.53
Create places for physical activity	17	8,567,076	503,946	6,190,972	1.38 (7.58)	0.22	30.74

Abbreviations: CPPW, Communities Putting Prevention to Work.

a Reach data were unreliable for some community interventions. Intervention costs without accompanying reach data were dropped from this table.

Costs per person reached tended to be lower for nutrition and physical activity interventions than for tobacco interventions (Table 1). For example, “Media to support improved nutrition to prevent obesity” had an average cost per person reached of $1.26. Across the 27 communities with reach estimates, costs were $0.33 to $10.14 per person reached. Similarly, for “Enhance access to healthy food retailer or healthier retail food, not transportation,” the average cost per person reached was $0.58, with a range across 22 communities of $0.03 to $8.24. Appendices C and D present cost estimates for all nutrition interventions.

The physical activity media intervention “Media to support improved physical activity to prevent obesity” had a low average cost per person reached of $0.76 across the 28 communities that implemented the intervention, but costs varied from $0.17 to $2,784.74 (Table 1). Although “Create places for physical activity” had a slightly higher average cost of $1.38, the cost was similar for all communities, ranging from $0.22 to $30.74 per person reached. Appendices E and F present cost estimates for all physical activity interventions.

We also examined costs per person reached for each MAPPS category (Table 2). Across all CPPW interventions with reach data, mean per person costs were highest for social support and services interventions. The next highest mean per person costs were for media interventions; lowest costs were for point of decision/promotion and price interventions. Of the CPPW tobacco interventions, social support and services interventions had the highest mean per person costs of about $3.00. Costs were generally lower for obesity interventions than for tobacco interventions. Per person costs were about $1 for obesity media and social support and services interventions and about half that ($0.38 to $0.61) for point of decision/promotion, access, and price interventions.

**Table 2:** MAPPS Category Costs per Person Reached, 2012 Dollars, 44 US Communities, 2010–2013

Summary Statistic	MAPPS Category, $				
	Media	Access	Point of Decision or Promotion	Price	Social Support and Services
**Overall**					
Mean^a^	1.10	0.61	0.40	0.43	1.62
Median	2.65	1.97	0.71	0.65	7.26
Minimum	0.16	0.11	0.06	0.01	0.13
Maximum	2,783.74	116.30	70.96	116.93	537.78
**Tobacco**					
Mean^a^	1.29	0.75	0.47	0.20	2.98
Median	2.76	1.63	1.21	0.22	4.16
Minimum	0.16	0.11	0.11	0.01	0.64
Maximum	153.84	115.37	70.96	14.11	537.78
**Obesity**					
Mean^a^	0.97	0.58	0.38	0.61	1.01
Median	2.49	2.00	0.53	1.08	10.22
Minimum	0.25	0.12	0.06	0.01	0.13
Maximum	2,783.74	116.30	5.79	116.93	86.21

Abbreviations: MAPPS, Media, Access, Point of decision/promotion, Price, Social support and services.

a The mean cost per person reached is weighted by the number of people reached.

**Figure 1:**
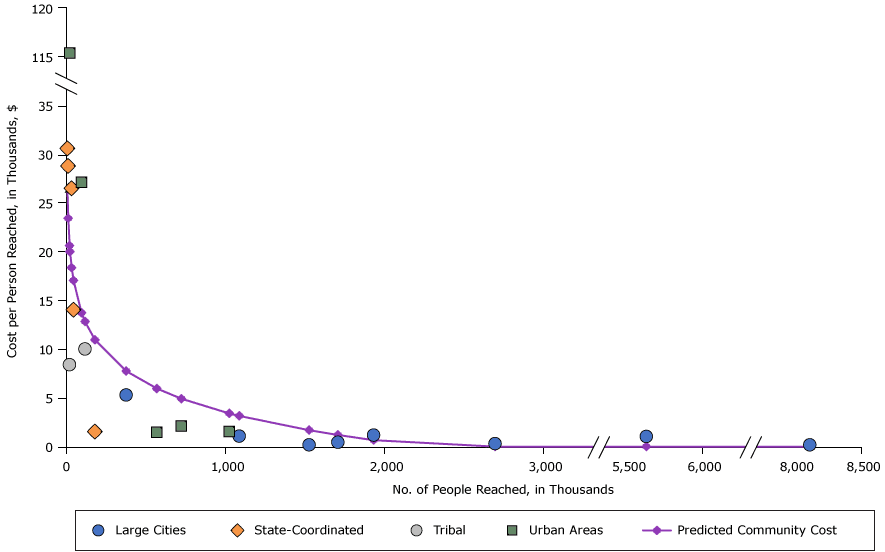
Costs per person reached for tobacco usage ban interventions, by intervention reach and community type, 2012 dollars. Abbreviations: NA, not applicable. **Table:** 

We report costs per person by intervention reach for selected tobacco, nutrition, and physical activity interventions. For all 3 interventions examined, we found that per person costs declined as intervention reach increased. Figure 1 shows estimated costs per person reached for “Usage bans.” Large cities had intervention reach ranging from 375,000 to over 8 million, and all but one large city had costs of less than $5 per person reached.

Community No.	Reach in thousands	Costs per Person, $				Predicted Cost per Person, $
		Large Cities	State-Coordinated	Tribal	Urban Areas	
Community 1	5	NA	30.58	NA	NA	26.30
Community 2	10	NA	28.79	NA	NA	23.42
Community 3	19	NA	NA	8.38	NA	20.61
Community 4	21	NA	NA	NA	115.37	20.00
Community 5	32	NA	26.50	NA	NA	18.36
Community 6	44	NA	14.02	NA	NA	17.06
Community 7	94	NA	NA	NA	27.13	13.73
Community 8	116	NA	NA	10.00	NA	12.83
Community 9	178	NA	1.53	NA	NA	10.95
Community 10	375	5.28	NA	NA	NA	7.74
Community 11	568	NA	NA	NA	1.44	5.95
Community 12	722	NA	NA	NA	2.10	4.91
Community 13	1,024	NA	NA	NA	1.51	3.40
Community 14	1,086	1.06	NA	NA	NA	3.14
Community 15	1,526	0.16	NA	NA	NA	1.67
Community 16	1,706	0.43	NA	NA	NA	1.19
Community 17	1,931	1.16	NA	NA	NA	0.66

**Figure 2:**
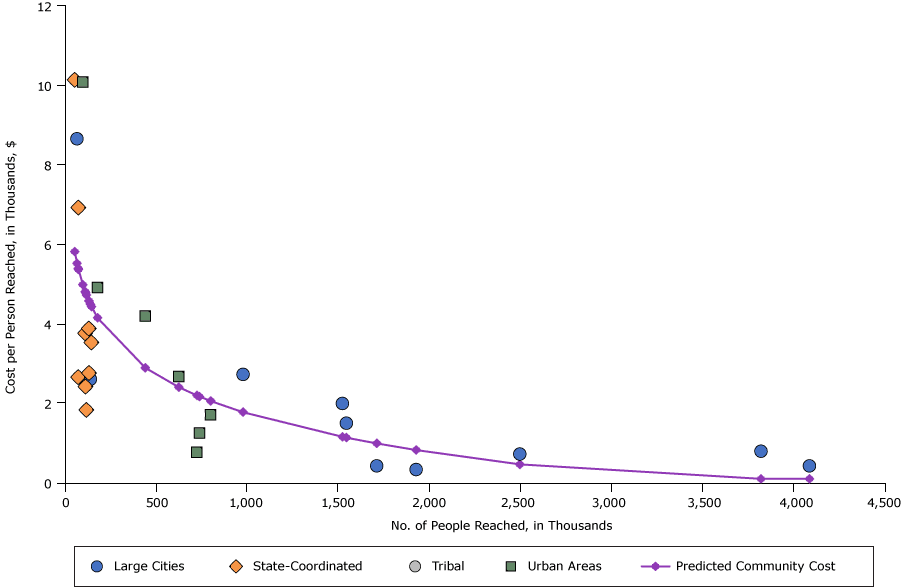
Costs per person reached for nutrition media interventions, by intervention reach and community type, 2012 dollars. Abbreviations: NA, not applicable; CPPW, Communities Putting Prevention to Work. **Table:** 

Figure 2 shows the distribution of costs per person reached for the intervention “Media to support improved nutrition to prevent obesity.” Despite being used in communities with intervention reach ranging from 55,000 to over 4 million, the estimated cost per person reached was less than $5 for most of the communities. In the communities where costs were highest, costs were $10 per person reached, indicating that, despite wide variation in the number of people reached by this intervention, costs per person reached were similar. All but one large city had costs of less than $3 per person reached.

Community No.	Reach in thousands	Costs per Person, $				Predicted Cost per Person, $
		Large Cities	State-Coordinated	Tribal	Urban Areas	
Community 1	76	NA	6.92	NA	NA	5.37
Community 2	443	NA	NA	NA	4.20	2.89
Community 3	116	NA	NA	2.46	NA	4.78
Community 4	142	2.60	NA	NA	NA	4.49
Community 5	4,091	0.42	NA	NA	NA	0.10
Community 6	181	NA	NA	NA	4.91	4.15
Community 7	802	NA	NA	NA	1.71	2.06
Community 8	100	NA	NA	NA	10.08	4.98
Community 9	55	NA	10.14	NA	NA	5.82
Community 10	115	NA	2.42	NA	NA	4.79
Community 11	741	NA	NA	NA	1.25	2.17
Community 12	120	NA	1.83	NA	NA	4.73
Community 13	2,501	0.72	NA	NA	NA	0.46
Community 14	133	NA	3.88	NA	NA	4.58
Community 15	148	NA	3.53	NA	NA	4.43
Community 16	727	NA	NA	NA	0.77	2.20
Community 17	627	NA	NA	NA	2.68	2.41
Community 18	3,825	0.79	NA	NA	NA	0.10
Community 19	113	NA	3.76	NA	NA	4.81
Community 20	1,526	2.00	NA	NA	NA	1.16
Community 21	980	2.73	NA	NA	NA	1.78
Community 22	1,715	0.42	NA	NA	NA	0.99
Community 23	1,548	1.50	NA	NA	NA	1.14
Community 24	1,931	0.33	NA	NA	NA	0.83
Community 25	68	8.66	NA	NA	NA	5.52
Community 26	135	NA	2.76	NA	NA	4.57
Community 27	75	NA	2.66	NA	NA	5.39

**Figure 3:**
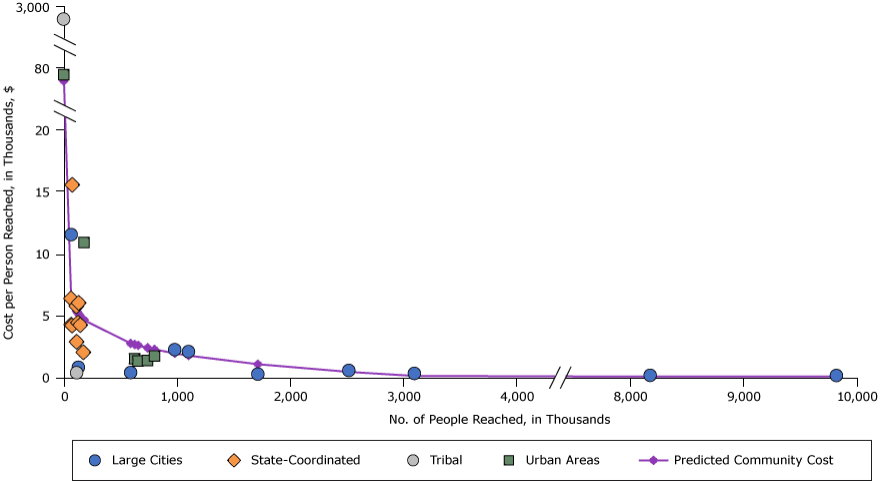
Costs per person reached for physical activity media interventions, by intervention reach and community type, 2012 dollars. Abbreviations: NA, not applicable; CPPW, Communities Putting Prevention to Work. **Table:** 

Figure 3 shows costs per person reached for the intervention “Media to support improved physical activity to prevent obesity.” Costs varied widely for this intervention; a tribal community with an estimated intervention reach of 160 people had the highest cost per person reached of almost $2,800. The next highest cost was for an urban area ($80). The other 26 communities had costs of $15 or less.

Community No.	Reach (in thousands)	Costs per Person, $				Predicted Cost per Person, $
		Large Cities	State-Coordinated	Tribal	Urban Areas	
Community 1	76	NA	15.59	NA	NA	6.04
Community 2	2	NA	NA	NA	79.98	79.98
Community 3	116	NA	NA	0.40	NA	5.37
Community 4	2,517	0.58	NA	NA	NA	0.49
Community 5	9,819	0.17	NA	NA	NA	0.10
Community 6	181	NA	NA	NA	10.94	4.66
Community 7	802	NA	NA	NA	1.75	2.30
Community 8	66	NA	6.40	NA	NA	6.26
Community 9	658	NA	NA	NA	1.35	2.61
Community 10	67	NA	4.29	NA	NA	6.24
Community 11	115	NA	2.90	NA	NA	5.38
Community 12	741	NA	NA	NA	1.38	2.42
Community 13	128	NA	4.44	NA	NA	5.21
Community 14	1,103	2.11	NA	NA	NA	1.79
Community 15	133	NA	6.04	NA	NA	5.15
Community 16	148	NA	4.26	NA	NA	4.98
Community 17	627	NA	NA	NA	1.54	2.69
Community 18	8,175	0.19	NA	NA	NA	0.10
Community 19	113	NA	5.78	NA	NA	5.40
Community 20	130	0.84	NA	NA	NA	5.19
Community 21	980	2.26	NA	NA	NA	1.98
Community 22	0	NA	NA	2,783.74	NA	2,783.74
Community 23	1,715	0.28	NA	NA	NA	1.09
Community 24	3,095	0.35	NA	NA	NA	0.16
Community 25	593	0.41	NA	NA	NA	2.78
Community 26	68	11.57	NA	NA	NA	6.21
Community 27	175	NA	2.05	NA	NA	4.72
Community 28	75	NA	4.22	NA	NA	6.06

## Discussion

The CPPW cost study was one of the first to use a systematic, prospective approach to estimate costs of evidence-based community interventions across multiple diverse communities. On average, half of CPPW costs (52%) went to partner organizations that worked with awardees, although partner contributions varied across communities. Because a condition of receiving a CPPW award was having a high level of readiness to implement interventions in a short time, we expected to see a high degree of partner support to supplement the more limited capacity of awardees. In-kind resources also were an important contribution to CPPW programs, as awardees were encouraged to use other resources to promote sustainability, including foundation funding, other US government funding sources, and state appropriations. On average, 6% of total CPPW costs were donations of labor or nonlabor contributions.

Intervention costs per person reached generally declined as the number of people reached increased. Although this finding is consistent with economies of scale, other factors cannot be ruled out for 2 reasons. First, the concept of economies of scale refers to how output changes with inputs (ie, per unit cost decreases as output increases), and measures of CPPW output besides intervention reach are not yet available. Second, the CPPW Funding Opportunity Announcement suggested upper limits on awards by community size, and actual awards appear to be driven by de facto funding caps. For example, many of the awards for large cities clustered around $15 million, although the large city populations ranged from 1 million to nearly 10 million. Large cities could have responded to the budget caps by focusing on a limited number of interventions, but such did not appear to be the case.

Estimated costs were less than $5 per person reached for 84% of tobacco interventions, 88% of nutrition interventions, and 89% of physical activity interventions. Additionally, costs per person reached were less than $1 for about one-half of interventions. Interventions with the highest costs per person reached were tobacco cessation services and subsidized memberships to recreational facilities. The costs required to implement these types of social support and services interventions are largely variable costs, which means that the cost to reach each person served is roughly constant, thus resulting in higher cost per person reached. In contrast, costs for access, point of decision or promotion, and price interventions are mostly fixed, which means they typically do not depend on the number of people reached, leading to lower per-person costs as more people are reached.

Other research on the costs of community health promotion interventions is limited. Although several studies analyzed population-based tobacco interventions, those studies reported results as costs per unit of outcome achieved (eg, life year gained) (10–15); thus, results are not comparable with our cost estimates. Wu et al (8) identified 3 studies that estimated costs for physical activity access interventions implemented outside the United States and found costs ranging from $5 to $137 per person.

A limitation of our analysis is that costs and reach were obtained at the level of the objective and not the intervention. To compare intervention costs, we assigned objective costs and reach to interventions. If an intervention was used to support multiple objectives in a community, we assigned the largest objective reach to avoid overstating reach. However, if different people were reached by each objective linked to an intervention, our approach understates intervention reach and overstates intervention costs. Nonetheless, this approach is conservative in that our intervention cost estimates probably provide an upper bound for intervention costs.

Another limitation is that CPPW reach estimates typically reflected the total number of people reached, even if those people were not smokers or obese. One exception is smoking cessation services, which were provided only to smokers. However, because CPPW interventions were largely focused on prevention for populations at high risk for obesity or tobacco use or both (16–20), our cost estimates may be interpreted as cost per at-risk person reached.

Our final limitation is that cost data were self-reported by community representatives. We verified quarterly total costs entered in the CSI against quarterly expenditures reported in ARRA financial reports; however, we could not verify the accuracy of allocations to objectives and strategies or in-kind contributions.

The initial study goals were 1) to determine how cost data for prevention activities could be collected, 2) to understand the challenges of collecting cost information, and 3) to build an accurate tool for cost data collection. An important challenge was collecting uniform cost data that could be aggregated and standardized across communities, given changes during the study in community activities. As more community prevention programs collect and report intervention costs, it will be important to compile and disseminate cost estimates to inform program design and provide input for cost-benefit evaluations.

## Conclusion

Although the CPPW program was implemented as one of many ARRA-funded strategies to create jobs, it also supported the adoption of community-based tobacco use and obesity prevention interventions in 44 US communities. Estimated costs to implement CPPW interventions are useful for planning for intervention implementation and evaluating costs relative to CPPW-related short- and long-term health outcomes.

## Appendices

This file is available for download as a Microsoft Word document.

Appendix A. Tobacco Intervention Cost Summary in 2012 Dollars, 44 US Communities, 2010–2013

Appendix B. Tobacco Intervention Cost per Person Reached Summary in 2012 Dollars, 44 US Communities, 2010–2013

Appendix C. Nutrition Intervention Cost Summary in 2012 Dollars, 44 US Communities, 2010–2013

Appendix D. Nutrition Intervention Cost per Person Reached Summary in 2012 Dollars, 44 US Communities, 2010–2013

Appendix E. Physical Activity Intervention Cost Summary in 2012 Dollars, 44 US Communities, 2010–2013

Appendix F. Physical Activity Intervention Cost per Person Reached Summary in 2012 Dollars, 44 US Communities, 2010–2013
