# Supplementary material for: Intervention Costs From Communities Putting Prevention to Work
Source: Prev Chronic Dis. 2016 Jul 28;13:E98. doi: 10.5888/pcd13.150368 (PMC4975201; doi:10.5888/pcd13.150368)
Supplement: Supplementary file 1 [file 15_0368_Appendices.docx]

Appendix A. Tobacco Intervention Cost Summary in 2012 Dollars, 44 US Communities, 2010–2013

| Intervention Description | N^a^ | Aggregate Cost, $ | Mean Community Cost, $ | Total No. of People Reached |
| --- | --- | --- | --- | --- |
| Usage bans | 20 | 26,566,358 | 1,328,318 | 25,976,876 |
| Counteradvertising | 14 | 35,433,968 | 2,530,998 | 22,550,786 |
| Cessation services — counseling or brief intervention | 10 | 7,680,253 | 768,025 | 2,335,652 |
| Cessation services — other | 10 | 11,190,978 | 1,119,098 | 2,175,929 |
| Restrict sales | 9 | 5,100,942 | 566,771 | 7,127,654 |
| Media and advertising restrictions consistent with federal law | 8 | 11,476,766 | 1,434,596 | 12,842,437 |
| Restrict point-of-purchase advertising as allowable under federal law | 8 | 2,210,532 | 276,317 | 5,879,485 |
| Point of purchase — other | 7 | 2,798,470 | 399,781 | 4,748,826 |
| Pricing strategy — restrict free samples | 7 | 1,398,064 | 199,724 | 1,336,152 |
| Cessation services — quitline, unspecified | 6 | 5,026,272 | 837,712 | 86,454 |
| Pricing strategy — fees | 6 | 1,504,616 | 250,769 | 13,356,015 |
| Cessation services — referral | 5 | 680,109 | 136,022 | 280,292 |
| Zoning restrictions (eg, outlet density) | 5 | 1,270,448 | 254,090 | 5,223,855 |
| Ban branded promotional items and prizes | 4 | $877,289 | 219,322 | 605,218 |
| Cessation event | 4 | 281,449 | 70,362 | 3,016,023 |
| Cessation services — quitline with NRT | 4 | 2,213,456 | 553,364 | 1,409,228 |
| Cessation services — screening | 4 | 1,479,433 | 369,858 | 539,718 |
| Policy enforcement — tobacco | 3 | 410,625 | 136,875 | 3,741,500 |
| Ban brand-name sponsorships | 2 | 154,445 | 77,223 | 380,131 |
| Cessation services — quitline without NRT | 2 | 1,065,008 | 532,504 | 26,602 |
| NRT distribution | 2 | 332,259 | 166,129 | 176,400 |
| Ban self-service displays and vending | 1 | 41,327 | 41,327 | 2,695,598 |
| Health education/event — tobacco | 1 | 906,205 | 906,205 | 303,448 |
| Media to change behavior | 1 | 16,692 | 16,692 | 899,387 |
| Media to support policy, systems and environmental change | 1 | 115,101 | 115,101 | 19,124 |

^a^ N = number of communities. Reach data were unavailable for some community interventions. Intervention costs without accompanying reach data have been dropped from this table.

Appendix B. Tobacco Intervention Cost per Person Reached Summary in 2012 Dollars, 44 US Communities, 2010–2013

| Intervention Description | Weighted Mean, $^a^ | Unweighted Mean, $ | Median (SD), $ | Minimum–Maximum, $ |
| --- | --- | --- | --- | --- |
| Usage bans | 1.02 | 13.85 | 1.81 (26.25) | 0.16–115.37 |
| Counteradvertising | 1.57 | 3.81 | 2.65 (3.78) | 0.16–14.11 |
| Cessation services — counseling or brief intervention | 3.29 | 50.64 | 3.43 (107.66) | 0.42–348.91 |
| Cessation services — other | 5.14 | 789.40 | 7.91 (2,448.29) | 0.33–7,757.20 |
| Restrict sales | 0.72 | 1.56 | 0.86 (1.73) | 0.39–5.81 |
| Media and advertising restrictions consistent with federal law | 0.89 | 25.99 | 3.23 (53.36) | 0.16–153.84 |
| Restrict point-of-purchase advertising as allowable under federal law | 0.38 | 10.73 | 1.33 (24.46) | 0.09–70.96 |
| Point of purchase — other | 0.59 | 3.27 | 1.21 (5.53) | 0.12–15.72 |
| Pricing strategy — restrict free samples | 1.05 | 3.61 | 1.74 (4.90) | 0.18–14.11 |
| Cessation services — Quitline, unspecified | 58.14 | 182.56 | 172.33 (120.77) | 13.73–348.91 |
| Pricing strategy — fees | 0.11 | 0.13 | 0.13 (0.11) | 0.01–0.30 |
| Cessation services — referral | 2.43 | 100.38 | 1.38 (153.19) | 0.64–348.91 |
| Zoning restrictions (eg, outlet density) | 0.24 | 1.61 | 0.54 (2.19) | 0.02–5.21 |
| Ban branded promotional items and prizes | 1.45 | 2.17 | 2.13 (2.01) | 0.37–4.04 |
| Cessation event | 0.09 | 3.46 | 0.44 (6.34) | 0.02–12.97 |
| Cessation services — quitline with NRT | 1.57 | 2.95 | 1.69 (3.27) | 0.64–7.78 |
| Cessation services — screening | 2.74 | 137.21 | 99.07 (167.67) | 1.80–348.91 |
| Policy enforcement — tobacco | 0.11 | 7.29 | 0.18 (12.47) | 0.00–21.70 |
| Ban brand-name sponsorships | 0.41 | 2.20 | 2.20 (2.60) | 0.36–4.04 |
| Cessation services — quitline without NRT | 40.03 | 712.10 | 712.10 (995.50) | 8.18–1,416.03 |
| NRT distribution | 1.88 | 80.38 | 80.38 (112.79) | 0.62–160.13 |
| Ban self-service displays and vending | 0.02 | 0.02 | 0.02 ( — ) | 0.02–0.02 |
| Health education/event — tobacco | 2.99 | 2.99 | 2.99 ( — ) | 2.99–2.99 |
| Media to change behavior | 0.02 | 0.02 | 0.02 ( — ) | 0.02–0.02 |
| Media to support policy, systems, and environmental change | 6.02 | 6.02 | 6.02 ( — ) | 6.02–6.02 |

Abbreviations; NRT, nicotine replacement therapy; SD, standard deviation.

^a^ Weights each community’s cost by community reach in calculating mean.

Appendix C. Nutrition Intervention Cost Summary in 2012 Dollars, 44 US Communities, 2010–2013

| Intervention Description | N^a^ | Aggregate Cost, $ | Mean Community Cost, $ | Total Number of People Reached |
| --- | --- | --- | --- | --- |
| Media to support improved nutrition to prevent obesity | 27 | 29,132,936 | 1,078,998 | 23,033,492 |
| Enhance access to healthy food retailer or healthier retail food, not transportation | 22 | 8,890,749 | 404,125 | 15,311,544 |
| Restrict availability of less healthy foods and beverages | 22 | 11,594,192 | 527,009 | 20,988,404 |
| Signage for healthy vs less healthy items | 19 | 6,634,044 | 349,160 | 7,408,975 |
| Wellness policy | 19 | 7,255,550 | 381,871 | 16,086,801 |
| Improve nutritional content through policies, guidelines, or standards | 17 | 7,832,872 | 460,757 | 11,313,657 |
| Change prices of healthier foods and beverages relative to the cost of less healthy foods | 16 | 6,701,974 | 418,873 | 12,129,776 |
| Breastfeeding — support breastfeeding through policy change and maternity care practices | 15 | 5,493,643 | 366,243 | 428,762 |
| Zoning/land use policies/joint use agreements (eg, for farmers markets/community gardens) | 15 | 6,145,005 | 409,667 | 9,037,140 |
| Procurement | 12 | 1,985,763 | 165,480 | 3,007,205 |
| Enhance usability of SNAP/WIC/ EBT at healthier food retailers | 11 | 1,804,246 | 164,022 | 4,316,799 |
| Systems or infrastructure changes to facilitate direct farm to institution food supplies | 10 | 2,609,845 | 260,985 | 1,100,526 |
| Healthy vending | 9 | 1,387,554 | 154,173 | 4,704,827 |
| Produce placement and attractiveness | 9 | 1,683,029 | 187,003 | 2,441,068 |
| Supporting local food production (eg, community gardens, school gardens, home gardens) | 8 | 1,618,841 | 202,355 | 3,486,854 |
| Incentives or price discounts for purchase of healthy foods when using SNAP/WIC/EBT | 6 | 577,018 | 96,170 | 632,430 |
| Menu labeling | 6 | 583,103 | 97,184 | 936,007 |
| Health education/event | 5 | 970,413 | 194,083 | 414,150 |
| Competitive foods | 4 | 298,485 | 74,621 | 258,117 |
| Incentives to offer healthier foods/choices | 4 | 340,746 | 85,186 | 1,433,263 |
| Enhanced access to tap water through environmental supports | 3 | 1,145,632 | 381,877 | 320,852 |
| Healthy meetings | 3 | 182,736 | 60,912 | 25,232 |
| Policy enforcement | 2 | 172,898 | 86,449 | 1,579,339 |
| Reduce sodium through purchasing actions, labeling initiatives, restaurant standards | 2 | 1,055,426 | 527,713 | 8,275,133 |
| Improving or providing low-cost transportation to healthier food venues | 1 | 50,977 | 50,977 | 49,707 |
| Information systems | 1 | 375,088 | 375,088 | 20,000 |

Abbreviations: EBT, electronic benefits transfer; SNAP, Supplemental Nutrition Assistance Program; WIC, Special Supplemental Nutrition Program for Women, Infants, and Children.

^a^ N = number of communities. Reach data were unavailable for some community interventions. Intervention costs without accompanying reach data have been dropped from this table.

Appendix D. Nutrition Intervention Cost per Person Reached Summary in 2012 Dollars, 44 US Communities, 2010–2013

| Intervention Description | Weighted Mean, $^a^ | Unweighted Mean, $ | Median (SD), $ | Minimum–Maximum, $ |
| --- | --- | --- | --- | --- |
| Media to support improved nutrition to prevent obesity | 1.26 | 3.19 | 2.60 (2.77) | 0.33**–**10.14 |
| Enhance access to healthy food retailer or healthier retail food, not transportation | 0.58 | 2.32 | 1.01 (2.42) | 0.03–8.24 |
| Restrict availability of less healthy foods and beverages | 0.55 | 8.60 | 3.97 (14.67) | 0.10–64.46 |
| Signage for healthy vs. less healthy items | 0.90 | 2.78 | 0.80 (5.67) | 0.07–24.46 |
| Wellness policy | 0.45 | 46.45 | 2.56 (149.89) | 0.00–656.51 |
| Improve nutritional content through policies, guidelines or standards | 0.69 | 54.92 | 4.24 (201.79) | 0.09–837.64 |
| Change prices of healthier foods and beverages relative to the cost of less healthy foods | 0.55 | 3.75 | 1.68 (4.75) | 0.01–16.65 |
| Breastfeeding — Support breastfeeding through policy change and maternity care practices | 12.81 | 38.93 | 15.72 (41.65) | 0.38–132.09 |
| Zoning/land use policies/joint use agreements (eg, for farmers markets/ community gardens) | 0.68 | 50.39 | 2.01 (111.61) | 0.03–424.72 |
| Procurement | 0.66 | 3.46 | 1.66 (5.09) | 0.11–17.92 |
| Enhance usability of SNAP/WIC/ EBT at healthier food retailers | 0.42 | 16.84 | 3.67 (26.19) | 0.11–65.20 |
| Systems or infrastructure changes to facilitate direct farm to institution food supplies | 2.37 | 11.45 | 2.63 (21.69) | 0.50–71.77 |
| Healthy vending | 0.29 | 3.85 | 2.54 (3.60) | 0.03–9.31 |
| Produce placement and attractiveness | 0.69 | 3.58 | 0.55 (7.93) | 0.15–24.53 |
| Supporting local food production (eg, community gardens, school gardens, home gardens) | 0.46 | 43.64 | 4.34 (71.19) | 0.05–200.60 |
| Incentives or price discounts for purchase of healthy foods when using SNAP/WIC/EBT | 0.91 | 10.89 | 0.4 (25.60) | 0.20–63.15 |
| Menu labeling | 0.62 | 1.73 | 1.28 (1.77) | 0.33–5.16 |
| Health education/event | 2.34 | 16.24 | 14.66 (13.61) | 1.85–34.44 |
| Competitive foods | 1.16 | 2.56 | 2.22 (2.19) | 0.47–5.34 |
| Incentives to offer healthier foods/choices | 0.24 | 0.75 | 0.22 (1.19) | 0.02–2.52 |
| Enhanced access to tap water through environmental supports | 3.57 | 33.81 | 4.72 (52.91) | 1.82–94.88 |
| Healthy meetings | 7.24 | 8.47 | 8.58 (3.08) | 5.34–11.49 |
| Policy enforcement | 0.11 | 2.07 | 2.07 (2.89) | 0.03–4.11 |
| Reduce sodium through purchasing actions, labeling initiatives, restaurant standards | 0.13 | 1.15 | 1.15 (1.48) | 0.10–0.19 |
| Improving or providing low-cost transportation to healthier food venues | 1.03 | 1.03 | 1.03 ( — ) | 1.03–1.03 |
| Information systems | 18.75 | 18.75 | 18.75 ( — ) | 18.75–18.75 |

Abbreviations: EBT, electronic benefits transfer; SNAP, Supplemental Nutrition Assistance Program; WIC, Special Supplemental Nutrition Program for Women, Infants, and Children.

^a^ Weights each community’s cost by community reach in calculating mean.

Appendix E. Physical Activity Intervention Cost Summary in 2012 Dollars, 44 US Communities, 2010–2013

| Intervention Description | N^a^ | Aggregate Cost | Mean Community Cost | Total Number of People Reached |
| --- | --- | --- | --- | --- |
| Media to support improved physical activity to prevent obesity | 28 | 24,482,982 | 874,392 | 32,417,636 |
| Environmental supports to promote walking and cycling and other physical activity | 20 | 13,025,401 | 651,270 | 23,178,552 |
| Infrastructure — urban design and land use policies (eg, complete streets) | 18 | 4,765,859 | 264,770 | 29,325,108 |
| Create places for physical activity | 17 | 8,567,076 | 503,946 | 6,190,972 |
| Safe routes to schools | 15 | 11,120,584 | 741,372 | 5,027,190 |
| PE/Physical activity requirement afterschool/ childcare | 12 | 2,131,669 | 177,639 | 486,712 |
| PE/Physical activity requirement schools | 12 | 10,621,831 | 885,153 | 1,941,054 |
| Signage for public transportation, bike lanes/boulevard | 12 | 1,546,561 | 128,880 | 6,891,154 |
| Joint use agreement | 10 | 2,778,513 | 277,851 | 4,149,011 |
| Neighborhood/district/ jurisdiction plans that support biking or walking | 10 | 4,541,498 | 454,150 | 9,814,938 |
| Reduced price for park/facility use | 10 | 2,177,016 | 217,702 | 4,866,013 |
| Activity groups | 9 | 2,449,330 | 272,148 | 11,014,788 |
| Signage for neighborhood destinations in walkable/mixed-use areas | 9 | 2,398,428 | 266,492 | 7,061,825 |
| Enhance personal safety in areas where persons are or could be physically active, not safe routes to school | 8 | 5,150,538 | 643,817 | 5,527,011 |
| Worksite physical activity programs | 8 | 1,138,817 | 142,352 | 1,887,188 |
| Infrastructure changes to support biking or walking | 7 | 1,453,412 | 207,630 | 3,432,833 |
| Restrict screen time in afterschool/d care | 5 | 657,550 | 131,510 | 241,438 |
| Health education/event | 4 | 992,692 | 248,173 | 1,061,413 |
| Point-of-decision prompts | 4 | 467,877 | 116,969 | 10,097,651 |
| Improve access to public transportation | 3 | $133,809 | $44,603 | 3,162,813 |
| Incentives for active transit | 3 | 8,085 | 2,695 | 1,082,032 |
| Subsidized memberships to recreational facilities | 3 | 1,936,458 | 645,486 | 83,406 |
| Health impact assessment or similar | 2 | 136,497 | 68,248 | 1,579,339 |
| Policy enforcement | 2 | 95,232 | 47,616 | 747,896 |
| Product distribution or distribution of supports to promote physical activity | 2 | 66,582 | 33,291 | 45,977 |
| Wellness policy | 2 | 49,834 | 24,917 | 168,654 |
| Information systems — physical activity | 1 | 375,088 | 375,088 | 20,000 |

^a^ N = communities. Reach data were unavailable for some community interventions. Intervention costs without accompanying reach data have been dropped from this table.

Appendix F. Physical Activity Intervention Cost per Person Reached Summary in 2012 Dollars, 44 US Communities, 2010–2013

| Intervention Description | Weighted Mean, $^a^ | Unweighted Mean, $ | Median (SD), $ | Minimum–Maximum, $ |
| --- | --- | --- | --- | --- |
| Media to support improved physical activity to prevent obesity | 0.76 | 105.57 | 2.19 (525.08) | 0.17**–**2,783.74 |
| Environmental supports to promote walking and cycling and other physical activity | 0.56 | 44.66 | 1.31 (124.87) | 0.02**–**461.53 |
| Infrastructure — urban design and land use policies (eg, complete streets) | 0.16 | 0.57 | 0.29 (0.66) | 0.01**–**2.79 |
| Create places for physical activity | 1.38 | 4.43 | 1.45 (7.58) | 0.2**–**30.74 |
| Safe routes to schools | 2.21 | 14.89 | 10.58 (16.23) | 0.33**–**58.07 |
| PE/physical activity requirement afterschool/childcare | 4.38 | 20.92 | 10.24 (25.68) | 0.34**–**85.32 |
| PE/Physical activity requirement schools | 5.47 | 27.22 | 12.89 (31.01) | 1.56**–**95.95 |
| Signage for public transportation, bike lanes/boulevard | 0.22 | 1.29 | 0.19 (1.85) | 0.03**–**5.79 |
| Joint use agreement | 0.67 | 2.26 | 1.41 (2.97) | 0.06**–**10.18 |
| Neighborhood/district/jurisdiction plans that support biking or walking | 0.46 | 14.98 | 0.92 (44.52) | 0.06**–**141.67 |
| Reduced price for park/facility use | 0.45 | 1.18 | 0.73 (1.65) | 0.00**–**5.61 |
| Activity groups | 0.22 | 16.98 | 6.50 (19.65) | 0.06**–**54.92 |
| Signage for neighborhood destinations in walkable/mixed-use areas | 0.34 | 0.75 | 0.34 (1.00) | 0.03**–**2.92 |
| Enhance personal safety in areas where people are or could be physically active, not safe routes to school | 0.93 | 9.61 | 0.44 (24.88) | 0.06**–**71.15 |
| Worksite physical activity programs | 0.60 | 7.01 | 2.03 (10.19) | 0.16**–**30.08 |
| Infrastructure changes to support biking or walking | 0.42 | 0.44 | 0.20 (0.52) | 0.01**–**1.28 |
| Restrict screen time in afterschool/d care | 2.72 | 25.08 | 7.55 (42.37) | 0.73**–**100.17 |
| Health education/event | 0.94 | 24.01 | 18.18 (28.00) | 0.81**–**58.89 |
| Point-of-decision prompts | 0.05 | 1.22 | 0.27 (2.07) | 0.04**–**4.30 |
| Improve access to public transportation | 0.04 | 3.77 | 0.91 (5.74) | 0.02**–**10.38 |
| Incentives for active transit | 0.01 | 0.33 | 0.06 (0.52) | 0.00**–**0.93 |
| Subsidized memberships to recreational facilities | 23.22 | 34.19 | 31.61 (19.59) | 16.02**–**54.94 |
| Health impact assessment or similar | 0.09 | 1.50 | 1.50 (2.09) | 0.03**–**2.98 |
| Policy enforcement | 0.13 | 6.07 | 6.07 (8.56) | 0.02**–**12.12 |
| Product distribution or distribution of supports to promote physical activity | 1.45 | 58.56 | 58.56 (82.56) | 0.18**–**116.93 |
| Wellness policy | 0.30 | 0.33 | 0.33 (0.25) | 0.15**–**0.51 |
| Information systems — physical activity | 18.75 | 18.75 | 18.75 ( — ) | 18.75**–**18.75 |

^a^ Weights each community’s cost by community reach in calculating mean.
